# Cardiac Arrest Caused by Multiple Recurrent Pulmonary Embolism

**DOI:** 10.1155/2011/425090

**Published:** 2011-11-24

**Authors:** Kjartan Eskjaer Hannig, Steen Elkjaer Husted, Erik Lerkevang Grove

**Affiliations:** Department of Cardiology, Aarhus University Hospital, Brendstrupgaardsvej 100, 8200 Aarhus, Denmark

## Abstract

Pulmonary embolism is a common condition with a high mortality. We describe a previously healthy 68-year-old male who suffered three pulmonary embolisms during a short period of time, including two embolisms while on anticoagulant treatment. This paper illustrates three important points. (1) The importance of optimal anticoagulant treatment in the prevention of pulmonary embolism reoccurrence. (2) The benefit of immediate accessibility to echocardiography in the handling of haemodynamically unstable patients with an unknown underlying cause. (3) Thrombolytic treatment should always be considered and may be life-saving in patients with cardiac arrest suspected to be caused by pulmonary embolism.

## 1. Introduction

Pulmonary embolism (PE) is a common cardiovascular emergency often caused by deep vein thrombosis in the lower extremities [1, 2]. Three-month mortality in hospitalised patients with PE is almost 20% [3] and is especially high in patients with compromised circulation; in these patients thrombolysis should be considered [2, 4]. Furthermore, unfractionated or low-molecular-weight heparin (LMWH) is used for at least five days, and, simultaneously, oral anticoagulation with a vitamin K antagonist (VKA) is initiated. LMWH is stopped when the international normalised ratio (INR) has been within the therapeutic level of 2.0-3.0 for at least two days [2, 5]. The risk of recurrent venous thromboembolism (VTE) is highest during the first 6 to 12 months, but oral VKA can be expected to decrease recurrence rate to about 1% per year [5]. The duration of VKA treatment is usually 3–12 months and is based on an individual estimate of recurrence and bleeding risk [4].

## 2. Case Report

A previously healthy 68-year-old employed, non-smoking 62 kg male was admitted with worsening shortness of breath during one day. Nine weeks earlier, the patient had been admitted with cardiac arrest caused by a central PE, which was surgically removed. The patient had been discharged with VKA treatment (warfarin), and since then the INR had been between 2 and 3, as confirmed until nine days prior to the present admission, when the INR was 2.2. However, on admission the INR was only 1.7.

**Figure 1:**
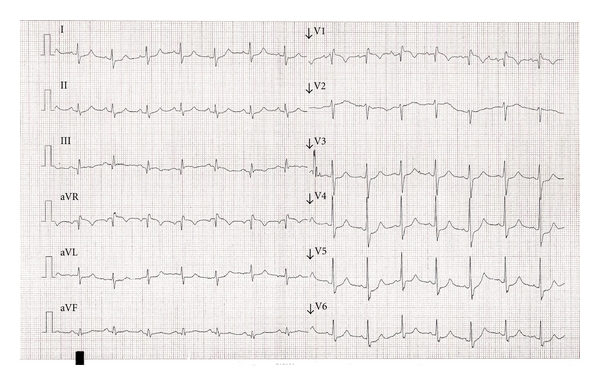
Electrocardiogram on admission.

The patient was in acute distress with a blood pressure of 60/40 mmHg and a peripheral saturation of 80% despite high-flow oxygen with a reservoir mask. Electrocardiography showed a discrete S_I_Q_III_T_III_ pattern and incomplete right bundle branch block (Figure 1). Acute echocardiography showed increased pulmonary pressure with tricuspid regurgitation with a pressure gradient of 50 mmHg. The patient received thrombolysis on suspicion of a new PE. Heparin infusion was initiated concurrently and was then changed to subcutaneous LMWH (dalteparin 6000 IU twice daily). A computed tomography (CT) scan raised suspicion of a malignant infiltrate in the left lung. The following day the patient had a fever (38.5°C) and a dry cough, and antibiotic treatment was started on suspicion of pneumonia. Five days after thrombolysis, echocardiography showed normalised pulmonary pressure.

**Figure 2:**
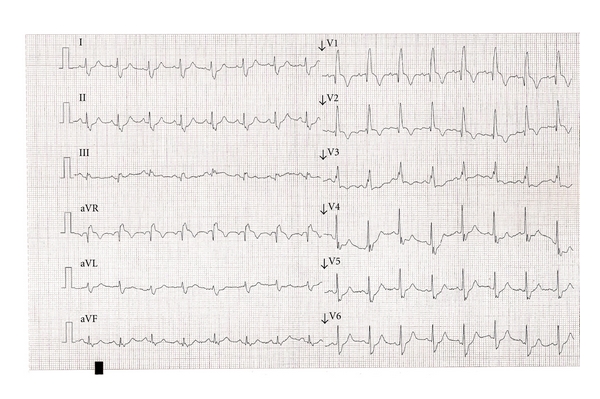
Electrocardiogram 1 week after admission.

One week after admission, the patient was started on broad-spectrum antibiotics due to persistent fever (38–38.5°C) and rising inflammatory markers. The patient had a near-syncope and now complained over worsening shortness of breath and a left-sided sharp, stabbing pain on breathing. The patient had a respiratory rate of 32, a peripheral saturation of 75% (3 litres oxygen flow), blood pressure was 80/50 mmHg, and the pulse was 105. An electrocardiogram showed sinus rhythm and a right bundle branch block, which was now complete (Figure 2). Blood gas analysis showed a fully compensated metabolic acidosis, no hypoxaemia, slight hypocapnia, and lactate 8.5 mmol/L. Treatment with high-flow oxygen and aggressive fluid resuscitation was initiated; this normalised the patient's symptoms, vital signs, and blood gas analysis during 15 minutes. However, after a few hours, the patient once again deteriorated resulting in cardiac arrest. Initially, the patient had pulseless electrical activity, and advanced resuscitation was initiated. After 15 minutes with nonshockable rhythms, resuscitation was stopped.

An autopsy showed that a massive central PE was the cause of death, and, additionally, cor pulmonale and a left-sided pulmonary infarction were found. Importantly, there were no signs of malignancy, deep venous thrombosis, or pneumonia.

## 3. Discussion

PE should be suspected in all patients who present with new or worsening dyspnoea, chest pain, or hypotension.

In haemodynamically stable patients, the first step is to assess the clinical probability of PE, which, for example, can be achieved with the Wells score [2]. In patients with low or intermediate clinical probability, a normal D-dimer practically rules out PE, whereas an increased D-dimer warrants further testing. Patients with a high clinical probability should undergo multidetector CT scan. A ventilation-perfusion scan is an alternative in patients allergic to contrast media or patients with renal failure.

In haemodynamically unstable patients, a multidetector CT scan should be performed. If this is not immediately available or if the patient's condition is too critical, an echocardiography should be performed. If signs of increased pulmonary pressure are observed, thrombolytic therapy may be considered according to guidelines [2, 6].

This case report illustrates three important points:

1. The importance of optimal anticoagulant treatment in the prevention of PE recurrence.
2. The benefit of immediate accessibility to echocardiography in the handling of haemodynamically unstable patients with an unknown underlying cause. In this case the presumably optimal anticoagulant treatment gave the wrong impression with regard to the underlying cause. An echocardiographic observation of recurrent increased pulmonary pressure would have suggested recurrent PE instead of severe septicaemia.
3. Thrombolysis should always be considered and may be life-saving in patients with cardiac arrest suspected to be caused by PE [7].

Risk factors for PE include reversible (surgery, immobilisation, and pregnancy/postpartum) and nonreversible (cancer and congenital or acquired thrombophilia) factors, but many cases are idiopathic/unprovoked [5, 8]. The risk of recurrence is low in provoked PE (reversible factors) but is moderate to high in patients with nonreversible factors, particularly in patients with idiopathic PE [9].

The patient's aggressive thrombogenicity suggested the existence of an underlying malignancy which was, however, ruled out by autopsy. There was no family history of VTE, and, if the patient had had an inherited thrombophilia, the patient's first VTE would most probably have occurred at a younger age. On the other hand, antiphospholipid syndrome cannot be definitively excluded as a possible cause of the recurrent PE [5, 9, 10]. Postmortem testing for thrombophilia was not performed because the patient did not have children, and a positive finding would not have had any direct consequences.

In the rare case of recurrent PE, despite adequate systemic anticoagulation, an underlying cause must be identified if possible, and the following treatment options considered.

1. Increase the target INR to 2.5–3.5, acknowledging that this may increase bleeding risk.
2. Insert an inferior vena cava filter permanently or a retrievable filter temporarily. Only a few widely accepted indications for this procedure exist, such as absolute contraindication to systemic anticoagulation and failure of systemic anticoagulation in case of acute proximal venous thrombosis [4]. Importantly, complications of inferior vena cava filters are common [2].
3. In a case with difficulty in achieving the target INR, the use of newer anticoagulants (dabigatran etexilate, apixaban, or rivaroxaban) or LMWH might be considered.
